# Combined intrapleural and intrabronchial injection of fibrin glue for closing alveolar pleural fistula: a case report

**DOI:** 10.1186/s13019-019-0987-7

**Published:** 2019-09-18

**Authors:** Alfonso Fiorelli, Italia Odierna, Daniele Scarano, Francesco Caronia, Andrea Failla, Mario Iannotti, Mario Santini, Caterina Pace

**Affiliations:** 1 Thoracic Surgery Unit, Università della Campania Luigi Vanvitelli, Piazza Miraglia, 2, I-80138 Naples, Italy; 2 Anaesthesiology and Intensive Care Unit, Umberto I Hospital, Nocera Inferiore, Salerno Italy; 3 Thoracic Surgery Unit, Ospedale Civico, Palermo, Italy; 4 General Surgery Unit, Istituto Oncologico Mediterraneo, Catania, Italy; 5 Anaesthesiology and Intensive Care Unit, Università della Campania Luigi Vanvitelli, Naples, Italy

**Keywords:** Persistent air leaks, Alveolar pleural fistula, Fibrin glue, Case report

## Abstract

**Background:**

The treatment of persistent air leak is a challenge. Herein, we reported the combined intrabronchial and intrapleural injection of fibrin glue using fiber bronchoscopy to seal off an alveolar pleura fistula developed following necrotizing pneumonia in high-risk patient.

**Case presentation:**

A 74-year-old man was intubated in emergency for acute ischemic stroke. Percutaneous dilatational tracheostomy was then performed, and 15 days later patient returned to spontaneous breathing. However, he developed alveolar pleural fistula following necrotizing pneumonia with persistent air leaks. The intrabronchial and intrapleural injection of fibrin glue using fiber bronchoscopy sealed off the alveolar pleura fistula after that other endoscopic treatments as bronchial valve and intrabronchial fibrin glue application had failed.

**Conclusions:**

Our strategy is safe and easy to reproduce. It represents an additional method in the armamentarium of the physicians for the management of PAL when all standard strategies are unfeasible or fail.

**Electronic supplementary material:**

The online version of this article (10.1186/s13019-019-0987-7) contains supplementary material, which is available to authorized users.

## Introduction

PAL is a frustrating clinical condition due to a pathological communication between the lung and pleural space [1]. It may be associated with significant morbidity, mortality and prolonged hospital stay; thus, an early resolution is desirable. In the years, conservative, surgical and endoscopic techniques have been reported to manage this pathological condition, but the best treatment is still debate [2, 3].

Herein, we reported a new approach as the intrabronchial and intrapleural injection of FG using fiber bronchoscopy to seal off APF developed following necrotizing pneumonia in high-risk patient. The procedure was successful after that other endoscopic treatments as bronchial valve and intrabronchial FG application had failed.

## Case presentation

**Fig. 1:**
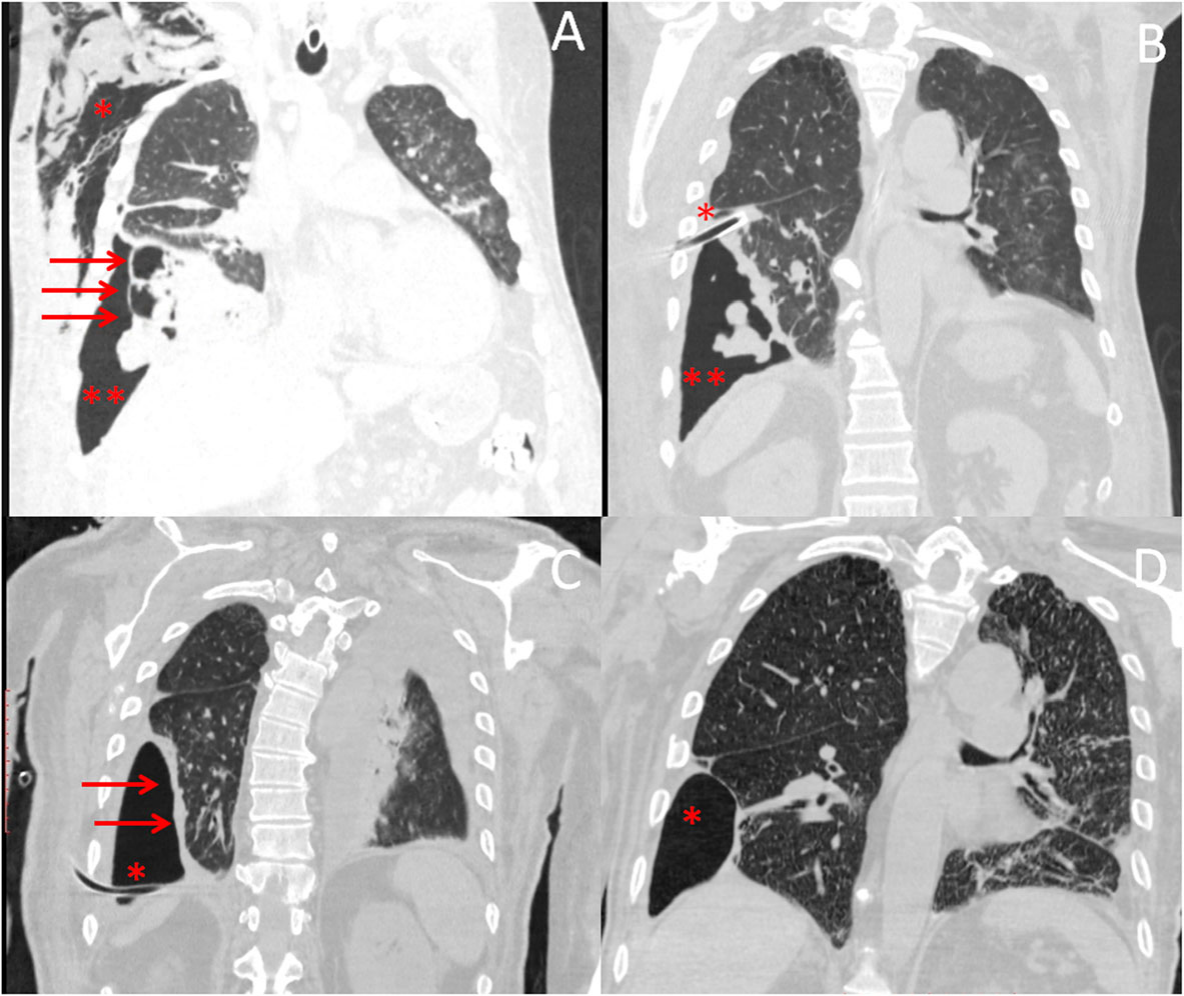
Chest computed tomography scan showed the presence of subcutaneous emphysema (*), pneumothorax (**), and necrotizing pneumonia with empyema (arrows) (Part **a**). After chest drainage placement (*), computed tomography scan showed the persistence of loculated pneumothorax (**) (Part **b**). Despite the insertion of chest tube (*), right lower lobe did not expand as it was trapped by pleural adhesions (arrows) (Part **c**). Following closure of alveolar pleura fistula, chest computed tomography showed no progression of loculated pneumothorax (*) (Part **d**)

**Fig. 2:**
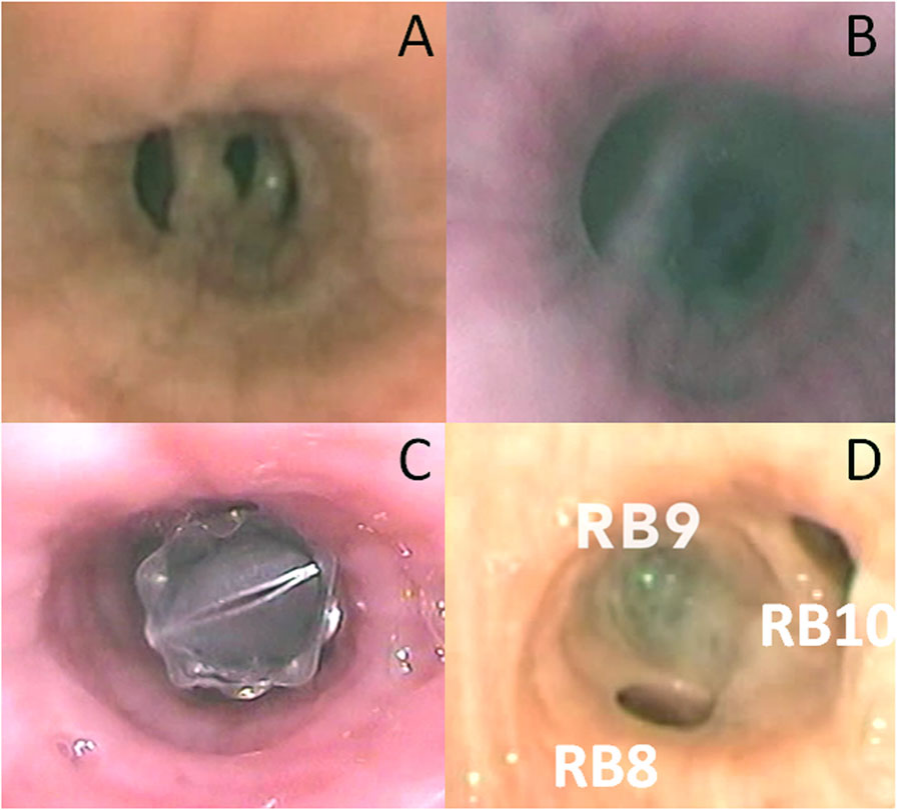
RB9 segment supplying the alveolar pleural fistula before (Part **a**) and after methylene blue injection (Part **b**). It was closed with endobronchial valve first (Part **c**), and then with fibrin glue (Part **d**)

A 74-year-old man was intubated in emergency for acute ischemic stroke, and then referred to Anesthesiology and Intensive Care Unit of our hospital. The patient’s medical history included cardiac disease and COPD. PDT was then performed, and patient returned to spontaneous breathing 15 days later. Despite systemic administration of broad-spectrum antibiotics (Vancomycin, Cefepime, and Azithromycin), he developed APF following necrotizing pneumonia that complicated with pneumothorax, empyema and subcutaneous emphysema (Fig. 1a). A 32 French tube was placed at the 5th intercostal space anterior axillar line with drainage of 1.500 l of brownish, putrid, and foul-swelling fluid. A negative suction (− 20 mmHg) was applied to chest drainage and daily chest X-ray showed the expansion of upper and middle lobe, but a loculated pneumothorax within lower lobe was seen on chest CT scan performed 15 days (Fig. 1b). A second 28 French chest tube was then placed using ultrasound as guide at the 8th intercostal space posterior axillar line with drainage of 500 mL of purulent material. The microbiological cultures of pleural fluid showed the presence of *Pseudomonas aeruginosa*; thus, ofloxacin (400 mg every 12 h) and ceftazidime (2 g every 8 h) were intravenously administered, in addition to clindamycin to provide empiric coverage against anaerobes and gram-positive cocci bacteria. The pleural space was also irrigated with 0.1% povidone-iodine solution (Betadine; 40 mL/h) until the eradication of pleural infection was obtained. However, the formation of dense adhesions trapped the lower lobe, and prevented its expansion (Fig. 1c); yet, the persistence of large bubbles in the drainage suspected the presence of APF. Methylene blue (1 ampoule diluted in 1-l saline solution) was injected via chest drainage into the pleural cavity, and was bronchoscopically identified within RB9 segment (Fig. 2a and b). The resolution of air leaks obtained by occluding the RB9 segment with an inflated-balloon catheter confirmed it to be the culprit segment. Thus, a Zephyr 5.5 EBV (Zephyr, PulmonX Corporation-Redwood City, CA, USA) was placed within RB9 segment (Fig. 2c) with temporary resolution of air leaks that recurred 4 days later due to valve dislocation. The valve was removed and the RB9 segment closed by intrabronchial injection of 10 mL of FG (Tisseel: Baxter Healthcare Corp, Deerfield, IL, USA) (Fig. 2d). The procedure was repeated twice at 1-week interval, but in both cases the fibrin clot dislocated.

**Fig. 3:**
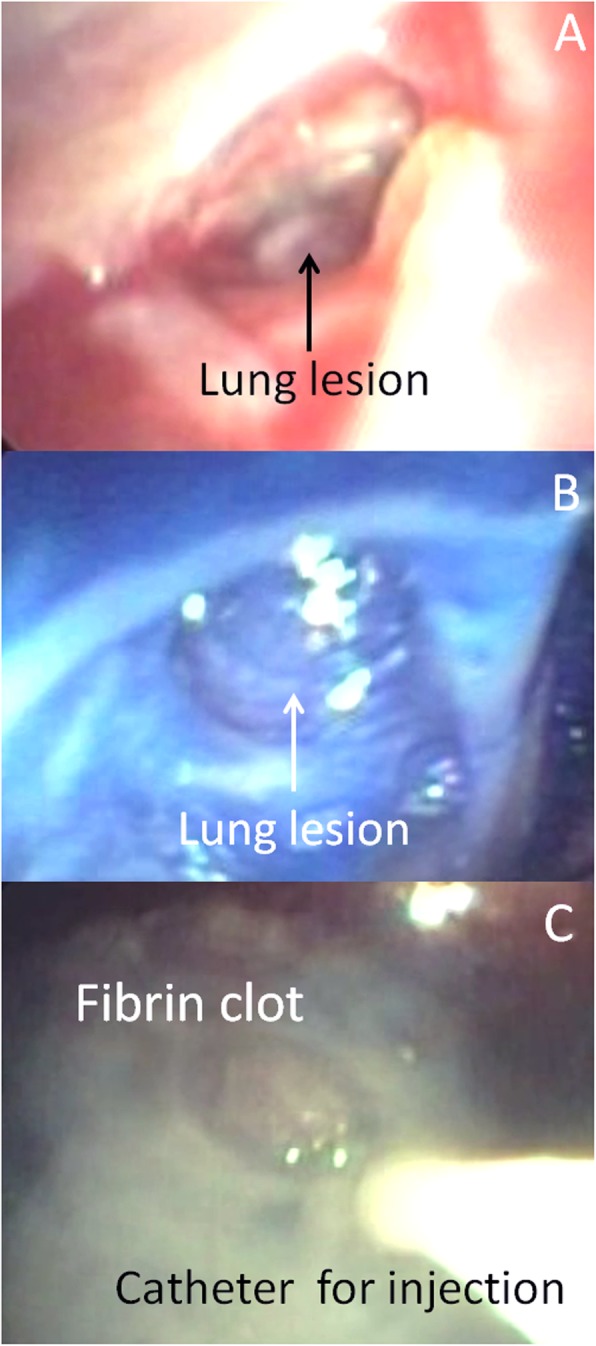
Alveolar pleura fistula (Part **a**) stained with methylene blue (Part **b**) after intrabronchial injection of blue solution within culprit bronchial segment. The lesion was repaired by intrapleural injection of fibrin glue using bronchoscopy (Part **c**)


Finally, fiber bronchoscopy, introduced through the chest drainage, explored the pleural cavity and showed a small APF (Fig. 3a) that was marked by methylene blue following intrabronchial injection of the blue solution within RB9 segment (Fig. 3b). The APF was filled by 20 mL of FG using a dedicated double-lumen catheter introduced through the working channel of fiber bronchoscopy (Fig. 3c). Furthermore, the RB9 segment was occluded by intrabronchial injection of 10 mL of FG. The chest drainage was then clamped, and a bronchial blocker was left with the balloon inflated within intermedius bronchus to prevent the intrapleural, and intrabronchial dislocation of fibrin clot, respectively. Two days later, the bronchial blocker was deflated, and the chest drainage opened. No recurrence of air-leaks occurred; drainage of non-purulent fluid was < 100 mL/24 h; chest CT scan showed no evidence of worsening pneumothorax, and of progressive subcutaneous emphysema; thus, chest tube was removed (Fig. 1d). Patient was then transferred to a rehabilitation center. He died 11 months later for cardiac failure. The entire procedure was summarized in Additional file 1: Video S1.

**Additional file 1:** **Video S1.** Video edited the main steps of the treatment including: (i) the identification of the culprit bronchial segment (RB9); (ii) closure with endobronchial valve and with fibrin glue; (iii) and the combined intrabronchial and intrapleural injection of fibrin glue to seal off alveolar pleura fistula. (MP4 159054 kb)


## Conclusions

The treatment of the present case was particularly challenging. Conservative treatment with chest drainage failed to resolve air leaks since dense pleural adhesions trapped the right lower lobe, and prevented its expansion. On the other hand, the presence of several pre-operative morbidities and poor patient’s clinical condition made unfeasible standard surgical repair with right lung exclusion. Thus, different endoscopic techniques were employed to treat APF. First, the culprit segment was closed with EBV [1–3] that dislocated 4 days later. Despite we used the largest valve commercially available (5.5 Zephyr EBV), probably it was too small for the size of the RB9 segment. Yet, the loss of elastic recoil related to underlying COPD increased the risk of dislocation [4]. Second, we closed the RB9 segment with FG [2, 3], but also this procedure failed as fibrin clot dislocated probably due to frequent airway aspirations.

Thus, we planned a personalized approach, not been reported before, as the combined intrapleural and intrabronchial injection of FG using fiber bronchoscopy. Kinoshita et al. [5], and Shrestha et al. [6] previously treated PAL by intrapleural injection of FG via a chest tube. In both cases, the FG was blindly injected within pleural cavity, while, in the present, the APF was sealed off under bronchoscopic view. In the same setting, FG was also injected within RB9 segment and a balloon-inflated catheter was temporarily left in the intermedius bronchus to avoid the dislodgement of fibrin clot, and to preserve the ventilation of right upper lobe.

Our procedure should be applied in high selected cases. In patients under mechanical ventilation, the presence of a trapped lung is needed for the bronchoscopic insertion and the exploration of pleural cavity. Furthermore, the eradication of empyema should be obtained before the intrapleural application of FG. The fibrin clot is a foreign body degraded 14 days after its formation [2, 3], and, in theory, it may prevent the resolution of infection. From a technically point of view, we recommend: (i) the use of large chest tube (≥ 28 French) to make easy the insertion and the handle of the fiber bronchoscopy; (ii) the careful exploration of pleural cavity to prevent additional injury of frail parenchyma; and (iii) the use of a dedicated double lumen catheter (Duplocath 180, Baxter AG, Vienna) for FG application. The two components of FG (fibrinogen and thrombin) should be simultaneously injected to avoid that a premature clotting occludes the catheter. Yet, during the procedure the tip of catheter should be distant from bronchoscopy to prevent any damage of working channel and/or the optic of the bronchoscopy. Thoracoscopy in awake patient may be a valuable alternative to our procedure as it allows to identify the AFP and to close it easily. However, thoracoscopy is performed in operation room, and may require a large incision, while the bronchoscopy is inserted via the chest drainage at patient’s bed side.

In closure, our strategy is safe and easy to reproduce. It represents an additional method in the armamentarium of the physicians for the management of PAL when all standard strategies are unfeasible or fail. Obviously, our impression should be corroborated by future, large experiences.

## Data Availability

The materials described in the manuscript, including all relevant raw data, will be freely available to any scientist wishing to use them for non-commercial purposes, without breaching participant confidentiality.

## Abbreviations

APF: Alveolar Pleural Fistula
COPD: Chronic Obstructive Pulmonary Disease
CT: Computed Tomography
EBV: Endobronchial valve
FG: Fibrin Glue
PAL: Persistent air leak
PDT: percutaneous dilatational tracheostomy
RB9: Right B9
